# 4-Nitro­phenyl 4-hy­droxy-3-methyl­benzoate

**DOI:** 10.1107/S1600536812048271

**Published:** 2012-11-30

**Authors:** S. Sreenivasa, H. C. Devarajegowda, H. T. Srinivasa, Vijith Kumar, B. S. Palakshamurthy

**Affiliations:** a Department of Studies and Research in Chemistry, Tumkur University, Tumkur 572 103, Karnataka, India; b Department of Physics, Yuvaraja’s College (Constituent College), University of Mysore, Mysore 570 005, Karnataka, India; c Raman Research Institute, C. V. Raman Avenue, Sadashivanagar, Bangalore 560 080, Karnataka, India; d Soild State and Structural Chemistry Unit, Indian Institute of Science, Bangalore 560 012, Karnataka, India

## Abstract

The asymmetric unit of the title compound, C_14_H_11_NO_5_, contains two independent mol­ecules in which the dihedral angles between the benzene rings are 89.27 (16) and 77.14 (12)°. In the crystal, mol­ecules are linked by O—H⋯O hydrogen bonds, generating *C*(8) chains propagating in [010] for one mol­ecule and [001] *C*(8) chains for the other. The chains are connected by C—H⋯O hydrogen bonds and π–π inter­actions [shortest centroid–centroid distance = 3.5908 (12)°], generating a three-dimensional network.

## Related literature

For general background to aromatic nitro groups, see: Ghosh *et al.* (2012 ▶); Sugiyama *et al.* (2002 ▶).
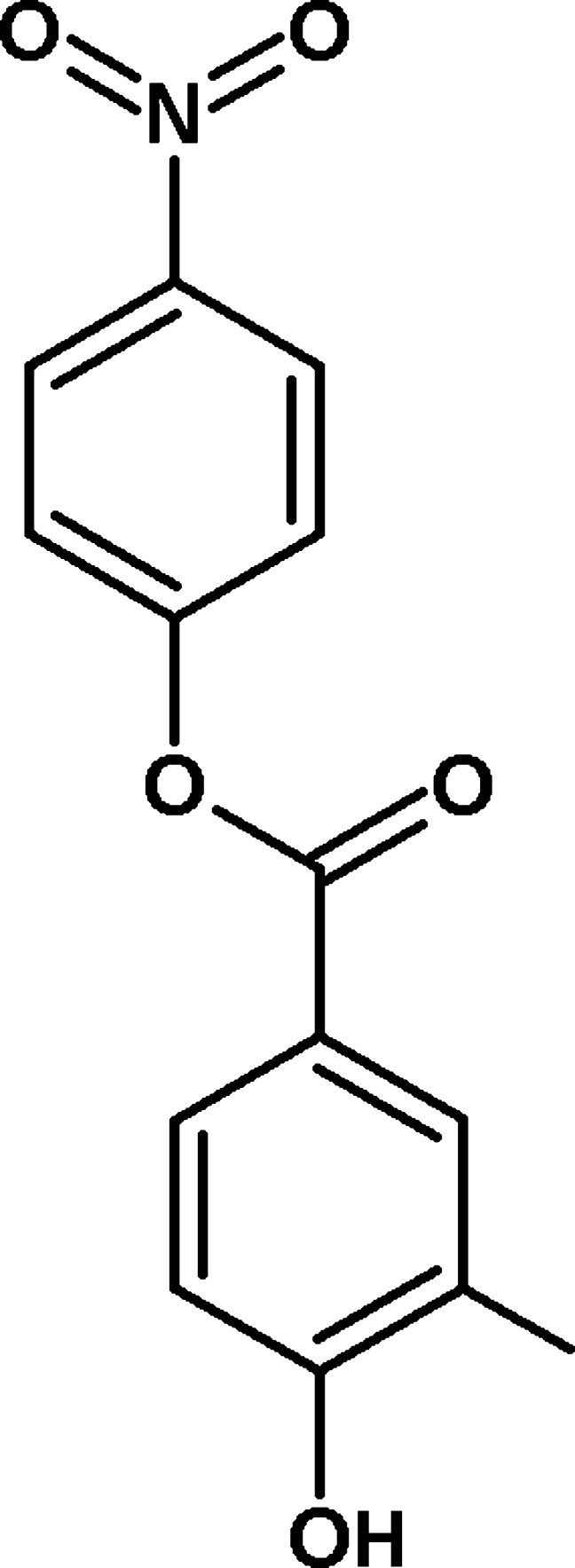


## Experimental

### 

#### Crystal data

C_14_H_11_NO_5_
*M* *_r_* = 273.24
Monoclinic, 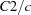

*a* = 42.313 (6) Å
*b* = 8.0047 (11) Å
*c* = 16.1078 (18) Å
β = 105.819 (4)°
*V* = 5249.2 (12) Å^3^
*Z* = 16
Mo *K*α radiation
μ = 0.11 mm^−1^
*T* = 298 K
0.24 × 0.20 × 0.16 mm

#### Data collection

Bruker SMART CCD diffractometer
Absorption correction: ψ scan (*SADABS*; Sheldrick, 2007 ▶) *T*_min_ = 0.975, *T*_max_ = 0.983
28460 measured reflections
4602 independent reflections
3349 reflections with *I* > 2σ(*I*)
*R*_int_ = 0.054

#### Refinement

*R*[*F* ^2^ > 2σ(*F* ^2^)] = 0.047
*wR*(*F* ^2^) = 0.143
*S* = 1.02
4602 reflections
361 parameters
H-atom parameters constrained
Δρ_max_ = 0.26 e Å^−3^
Δρ_min_ = −0.20 e Å^−3^

### 

Data collection: *SMART* (Bruker, 2001 ▶); cell refinement: *SAINT* (Bruker, 2001 ▶); data reduction: *SAINT*; program(s) used to solve structure: *SHELXS97* (Sheldrick, 2008 ▶); program(s) used to refine structure: *SHELXL97* (Sheldrick, 2008 ▶); molecular graphics: *ORTEP-3* (Farrugia, 2012 ▶); software used to prepare material for publication: *SHELXL97*.

## Supplementary Material

Crystal structure: contains datablock(s) I, global. DOI: 10.1107/S1600536812048271/hb6999sup1.cif

Structure factors: contains datablock(s) I. DOI: 10.1107/S1600536812048271/hb6999Isup2.hkl

Supplementary material file. DOI: 10.1107/S1600536812048271/hb6999Isup3.cml

Additional supplementary materials:  crystallographic information; 3D view; checkCIF report

## Figures and Tables

**Table 1:** Hydrogen-bond geometry (Å, °)

*D*—H⋯*A*	*D*—H	H⋯*A*	*D*⋯*A*	*D*—H⋯*A*
O1*A*—H1*A*⋯O2*A* ^i^	0.82	1.94	2.753 (2)	172
O1*B*—H1*B*⋯O2*B* ^ii^	0.82	1.94	2.727 (3)	160
C7*B*—H7*B*1⋯O4*A* ^iii^	0.96	2.50	3.418 (3)	159
C12*A*—H12*A*⋯O2*A* ^i^	0.93	2.54	3.245 (2)	132
C19*B*—H19*B*⋯O5*A* ^iv^	0.93	2.58	3.476 (4)	163

Symmetry codes: (i) 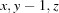; (ii) 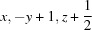; (iii) 
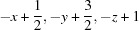
; (iv) 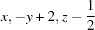.

**Fig. 1.**
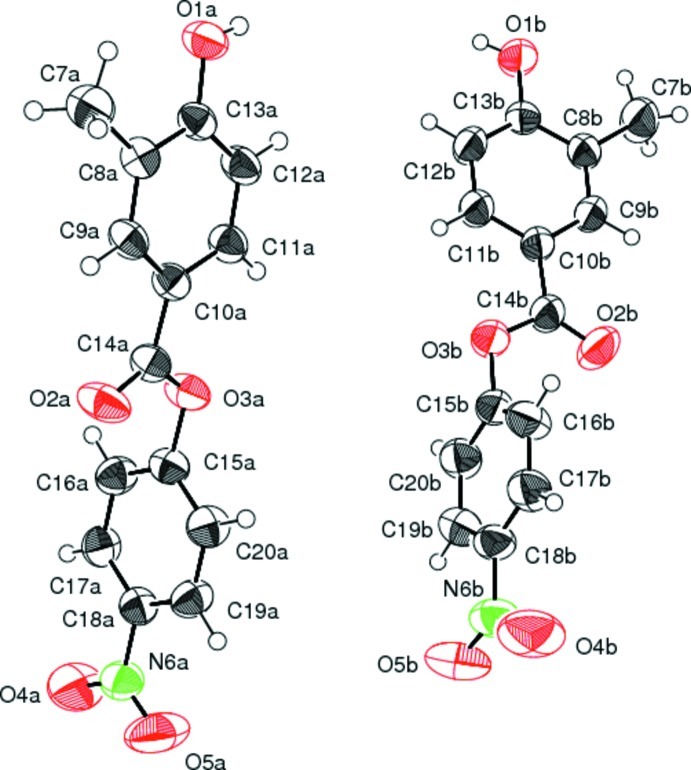
The molecular structure of the title compound. Displacement ellipsoids are drawn at the 50% probability level.

**Fig. 2.**
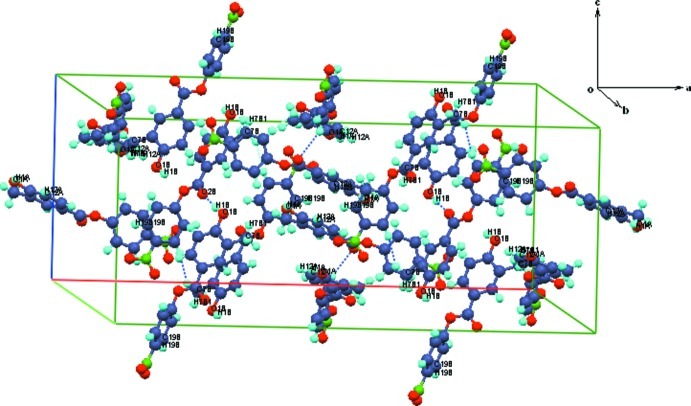
The packing of molecules.

## supplementary crystallographic information

### Comment

Electron withdrawing nitro group is an important structural element having good synthetic value in organic synthesis to achieve a wide variety of natural and biological active molecules (Ghosh *et al.*, 2012). Formation of two-component molecular crystals from nitro benzoic acid and aromatic or heterocyclic bases leads to discovery of new functional solid materials for nonlinear optics (Sugiyama *et al.*, 2002).

The asymmetric unit of 4-Nitrophenyl 4-hydroxy-3-methylbenzoate crystallographically two independent molecules are shown in Fig.1. Each independent molecule (a and b) is approximately perpendicular to each other; the dihedral angle is 89.17 (14)°. The dihedral angles between the benzene rings in the two molecules [(C8a–C13*a* and C15*a*–C20*a*) and C8b–C13*b* and C15*b*–C20*b*)] are 89.27 (16)° and 77.14 (12)° respectively. The crystal structure features O—H···O and C—H···O interactions (Table 1) and π—π interactions.

### Experimental

A mixture of 4-nitrophenol (0.100 g, 0.072 mol) and 4-hydroxy-3-methylbenzoic acid (0.109 g, 0.072 mol) and dicyclohexyldicarbodimide (0.150 g, 0.11 mol) in 1 ml of dry dimethylsufoxide (DMSO) was irradiated under microwave (600 MHz) for 5 *x* 60sec. Reaction was monitored by TLC, after completion of reaction; the reaction mixture was poured into ice cold, dilute hydrochloric acid which precipitated as ester. The crude product was filtered through Buckner funnel with vacuum, washed with water, crude precipitate was stirred for 1 hr with a saturated sodium bicarbonate solution to remove excess acid. The ester was again collected by filtration, washed repeatedly with water, air dried, and then repeatedly crystallized to get colourless plates from an ethanol-chloroform solvent mixture.

### Refinement

All H atoms were positioned geometrically, with O—H = 0.82, C—H = 0.93 Å for aromatic H, and C—H = 0.96 Å for methyl H,and refined using a riding model with *U*_iso_(H) = 1.5*U*_eq_(C) for methyl H and *U*_iso_(H) = 1.2*U*_eq_(C) for all other H.

### Crystal data

**Table:**

C_14_H_11_NO_5_	*F*(000) = 2272
*M**_r_* = 273.24	*D*_x_ = 1.383 Mg m^−^^3^
Monoclinic, *C*2/*c*	Melting point: 453 K
Hall symbol: -C 2yc	Mo *K*α radiation, λ = 0.71073 Å
*a* = 42.313 (6) Å	Cell parameters from 4602 reflections
*b* = 8.0047 (11) Å	θ = 2.0–25.1°
*c* = 16.1078 (18) Å	µ = 0.11 mm^−^^1^
β = 105.819 (4)°	*T* = 298 K
*V* = 5249.2 (12) Å^3^	Plate, colourless
*Z* = 16	0.24 × 0.20 × 0.16 mm

### Data collection

**Table:**

Bruker SMART CCD diffractometer	4602 independent reflections
Radiation source: fine-focus sealed tube	3349 reflections with *I* > 2σ(*I*)
Graphite monochromator	*R*_int_ = 0.054
ω and φ scans	θ_max_ = 25.1°, θ_min_ = 2.0°
Absorption correction: ψ scan (*SADABS*; Sheldrick, 2007)	*h* = −49→50
*T*_min_ = 0.975, *T*_max_ = 0.983	*k* = −9→9
28460 measured reflections	*l* = −19→19

### Refinement

**Table:**

Refinement on *F*^2^	Primary atom site location: structure-invariant direct methods
Least-squares matrix: full	Secondary atom site location: difference Fourier map
*R*[*F*^2^ > 2σ(*F*^2^)] = 0.047	Hydrogen site location: inferred from neighbouring sites
*wR*(*F*^2^) = 0.143	H-atom parameters constrained
*S* = 1.02	*w* = 1/[σ^2^(*F*_o_^2^) + (0.0757*P*)^2^ + 2.2456*P*] where *P* = (*F*_o_^2^ + 2*F*_c_^2^)/3
4602 reflections	(Δ/σ)_max_ < 0.001
361 parameters	Δρ_max_ = 0.26 e Å^−^^3^
0 restraints	Δρ_min_ = −0.20 e Å^−^^3^

### Special details

**Table:**

Experimental. IR (cm^-1^); 2923, 2851, 1739, 1603, 1513, 1246, 1199;. 1*H*-NMR (400 MHz, CDCl_3_):8.27 (d, 2H, J = 8.56 Hz, Ar—H), 7.79 (m, 2H, Ar—H), 7.43 (m, 2H, Ar—H), 6.78 (m, 1H, Ar—H), 5.01 (s, 1H, Ar—OH), 2.34 (t, 3H, J = 6.5 Hz, Ar—CH3); Elemental analysis: C_14_H_11_NO_5_ requires C, 61.54; H, 4.06; N, 5.13; found C, 61.95;H, 4.34; N, 4.79.
Geometry. All s.u.'s (except the s.u. in the dihedral angle between two l.s. planes) are estimated using the full covariance matrix. The cell s.u.'s are taken into account individually in the estimation of s.u.'s in distances, angles and torsion angles; correlations between s.u.'s in cell parameters are only used when they are defined by crystal symmetry. An approximate (isotropic) treatment of cell s.u.'s is used for estimating s.u.'s involving l.s. planes.
Refinement. Refinement of *F*^2^ against ALL reflections. The weighted *R*-factor *wR* and goodness of fit *S* are based on *F*^2^, conventional *R*-factors *R* are based on *F*, with *F* set to zero for negative *F*^2^. The threshold expression of *F*^2^ > 2σ(*F*^2^) is used only for calculating *R*-factors(gt) *etc*. and is not relevant to the choice of reflections for refinement. *R*-factors based on *F*^2^ are statistically about twice as large as those based on *F*, and *R*- factors based on ALL data will be even larger.

### Fractional atomic coordinates and isotropic or equivalent isotropic displacement parameters (Å^2^)

**Table:**

	*x*	*y*	*z*	*U*_iso_*/*U*_eq_	
O1A	−0.03888 (3)	−0.32059 (16)	0.40683 (10)	0.0550 (4)	
H1A	−0.0263	−0.3968	0.4036	0.082*	
O2A	0.00229 (4)	0.43257 (18)	0.37864 (11)	0.0652 (4)	
O3A	0.04796 (3)	0.29667 (16)	0.37629 (10)	0.0542 (4)	
O4A	0.12917 (6)	0.9576 (3)	0.45292 (13)	0.1007 (7)	
O5A	0.11233 (7)	0.9690 (3)	0.31718 (14)	0.1168 (9)	
N6A	0.11378 (5)	0.9012 (2)	0.38481 (14)	0.0611 (5)	
C7A	−0.07695 (5)	−0.0418 (3)	0.41356 (16)	0.0605 (6)	
H7A1	−0.0824	−0.1574	0.4175	0.091*	
H7A2	−0.0930	0.0096	0.3666	0.091*	
H7A3	−0.0767	0.0139	0.4665	0.091*	
C8A	−0.04364 (5)	−0.0287 (2)	0.39804 (12)	0.0432 (5)	
C9A	−0.02951 (5)	0.1231 (2)	0.38957 (12)	0.0446 (5)	
H9A	−0.0412	0.2204	0.3921	0.053*	
C10A	0.00168 (5)	0.1357 (2)	0.37741 (12)	0.0418 (4)	
C11A	0.01863 (5)	−0.0096 (2)	0.37014 (13)	0.0462 (5)	
H11A	0.0392	−0.0033	0.3598	0.055*	
C12A	0.00513 (5)	−0.1623 (2)	0.37818 (13)	0.0489 (5)	
H12A	0.0165	−0.2595	0.3729	0.059*	
C13A	−0.02537 (5)	−0.1723 (2)	0.39407 (12)	0.0423 (4)	
C14A	0.01605 (5)	0.3012 (2)	0.37702 (12)	0.0440 (5)	
C15A	0.06391 (5)	0.4505 (2)	0.37895 (13)	0.0462 (5)	
C16A	0.08228 (5)	0.5103 (3)	0.45681 (14)	0.0542 (5)	
H16A	0.0835	0.4515	0.5074	0.065*	
C17A	0.09898 (5)	0.6592 (3)	0.45916 (13)	0.0526 (5)	
H17A	0.1117	0.7023	0.5112	0.063*	
C18A	0.09643 (5)	0.7423 (3)	0.38305 (13)	0.0465 (5)	
C19A	0.07829 (5)	0.6819 (3)	0.30503 (13)	0.0530 (5)	
H19A	0.0771	0.7404	0.2544	0.064*	
C20A	0.06183 (5)	0.5328 (3)	0.30305 (14)	0.0544 (5)	
H20A	0.0495	0.4886	0.2509	0.065*	
O1B	0.29237 (4)	0.4579 (3)	0.43358 (10)	0.0803 (6)	
H1B	0.2789	0.4362	0.4602	0.120*	
O2B	0.24794 (5)	0.5330 (3)	0.02839 (11)	0.1045 (8)	
O3B	0.20070 (4)	0.4732 (2)	0.05604 (9)	0.0719 (5)	
O4B	0.13548 (7)	0.4123 (4)	−0.33906 (14)	0.1226 (9)	
O5B	0.11288 (6)	0.6376 (3)	−0.31360 (14)	0.1102 (8)	
N6B	0.13174 (6)	0.5217 (3)	−0.29087 (14)	0.0750 (6)	
C7B	0.33294 (6)	0.5292 (5)	0.33073 (18)	0.0921 (10)	
H7B1	0.3386	0.5163	0.3923	0.138*	
H7B2	0.3383	0.6403	0.3168	0.138*	
H7B3	0.3450	0.4497	0.3068	0.138*	
C8B	0.29669 (5)	0.4997 (3)	0.29354 (13)	0.0557 (6)	
C9B	0.28152 (5)	0.5074 (3)	0.20657 (14)	0.0582 (6)	
H9B	0.2942	0.5300	0.1689	0.070*	
C10B	0.24797 (5)	0.4827 (3)	0.17292 (13)	0.0515 (5)	
C11B	0.22927 (6)	0.4457 (3)	0.22887 (14)	0.0628 (6)	
H11B	0.2068	0.4271	0.2075	0.075*	
C12B	0.24386 (6)	0.4367 (4)	0.31560 (14)	0.0663 (7)	
H12B	0.2312	0.4119	0.3531	0.080*	
C13B	0.27708 (5)	0.4640 (3)	0.34785 (13)	0.0553 (5)	
C14B	0.23353 (6)	0.4987 (3)	0.08041 (14)	0.0615 (6)	
C15B	0.18461 (5)	0.4871 (3)	−0.03191 (14)	0.0592 (6)	
C16B	0.18584 (6)	0.3559 (3)	−0.08597 (16)	0.0668 (6)	
H16B	0.1981	0.2609	−0.0650	0.080*	
C17B	0.16863 (6)	0.3677 (3)	−0.17177 (15)	0.0654 (6)	
H17B	0.1693	0.2816	−0.2100	0.078*	
C18B	0.15043 (6)	0.5094 (3)	−0.19964 (14)	0.0591 (6)	
C19B	0.14870 (6)	0.6388 (3)	−0.14563 (16)	0.0646 (6)	
H19B	0.1360	0.7325	−0.1661	0.077*	
C20B	0.16627 (6)	0.6272 (3)	−0.06012 (15)	0.0647 (6)	
H20B	0.1657	0.7136	−0.0220	0.078*	

### Atomic displacement parameters (Å^2^)

**Table:**

	*U*^11^	*U*^22^	*U*^33^	*U*^12^	*U*^13^	*U*^23^
O1A	0.0550 (9)	0.0383 (7)	0.0738 (10)	−0.0022 (6)	0.0213 (8)	0.0009 (6)
O2A	0.0512 (9)	0.0370 (8)	0.1076 (13)	0.0056 (7)	0.0219 (9)	0.0054 (8)
O3A	0.0462 (8)	0.0380 (7)	0.0792 (10)	−0.0012 (6)	0.0183 (7)	0.0010 (7)
O4A	0.1216 (18)	0.0931 (14)	0.0767 (13)	−0.0565 (13)	0.0087 (12)	−0.0182 (11)
O5A	0.167 (2)	0.0962 (16)	0.0801 (14)	−0.0701 (16)	0.0215 (15)	0.0111 (11)
N6A	0.0621 (12)	0.0575 (11)	0.0652 (13)	−0.0157 (9)	0.0198 (10)	−0.0026 (10)
C7A	0.0465 (12)	0.0540 (13)	0.0842 (16)	−0.0002 (10)	0.0234 (12)	−0.0030 (11)
C8A	0.0384 (11)	0.0444 (11)	0.0441 (11)	0.0027 (8)	0.0068 (9)	−0.0019 (8)
C9A	0.0422 (11)	0.0360 (10)	0.0531 (12)	0.0072 (8)	0.0089 (9)	−0.0016 (8)
C10A	0.0418 (11)	0.0379 (10)	0.0429 (11)	0.0038 (8)	0.0068 (9)	0.0014 (8)
C11A	0.0419 (11)	0.0412 (11)	0.0563 (12)	0.0032 (8)	0.0149 (10)	−0.0017 (9)
C12A	0.0483 (12)	0.0353 (10)	0.0647 (13)	0.0063 (8)	0.0183 (10)	−0.0025 (9)
C13A	0.0448 (11)	0.0379 (10)	0.0403 (10)	−0.0009 (8)	0.0050 (9)	−0.0016 (8)
C14A	0.0408 (11)	0.0393 (10)	0.0486 (12)	0.0047 (9)	0.0069 (9)	0.0028 (8)
C15A	0.0390 (11)	0.0406 (10)	0.0591 (13)	−0.0001 (8)	0.0134 (10)	0.0009 (9)
C16A	0.0580 (13)	0.0565 (13)	0.0459 (12)	−0.0025 (10)	0.0105 (11)	0.0070 (10)
C17A	0.0548 (13)	0.0571 (13)	0.0420 (11)	−0.0066 (10)	0.0064 (10)	−0.0048 (9)
C18A	0.0410 (11)	0.0476 (11)	0.0508 (12)	−0.0037 (9)	0.0120 (9)	−0.0037 (9)
C19A	0.0543 (13)	0.0583 (13)	0.0443 (12)	−0.0088 (10)	0.0100 (10)	0.0053 (9)
C20A	0.0507 (13)	0.0594 (13)	0.0470 (12)	−0.0101 (10)	0.0029 (10)	−0.0025 (10)
O1B	0.0576 (10)	0.1384 (17)	0.0412 (9)	0.0057 (10)	0.0071 (8)	0.0077 (9)
O2B	0.0605 (11)	0.207 (3)	0.0457 (10)	−0.0223 (13)	0.0144 (9)	0.0125 (12)
O3B	0.0469 (9)	0.1225 (15)	0.0421 (9)	−0.0040 (9)	0.0049 (7)	0.0036 (9)
O4B	0.135 (2)	0.151 (2)	0.0596 (13)	0.0139 (17)	−0.0116 (13)	−0.0215 (14)
O5B	0.1202 (18)	0.1087 (17)	0.0772 (14)	0.0137 (15)	−0.0145 (13)	0.0291 (12)
N6B	0.0712 (15)	0.0898 (17)	0.0529 (13)	−0.0161 (13)	−0.0015 (11)	0.0063 (12)
C7B	0.0491 (15)	0.161 (3)	0.0627 (16)	−0.0108 (17)	0.0101 (12)	0.0000 (17)
C8B	0.0443 (12)	0.0739 (15)	0.0475 (12)	0.0047 (10)	0.0105 (10)	−0.0010 (10)
C9B	0.0485 (13)	0.0830 (16)	0.0463 (12)	0.0003 (11)	0.0183 (11)	0.0036 (11)
C10B	0.0441 (12)	0.0677 (14)	0.0423 (11)	0.0020 (10)	0.0114 (9)	−0.0009 (10)
C11B	0.0436 (12)	0.0952 (18)	0.0487 (13)	−0.0038 (12)	0.0112 (10)	0.0032 (12)
C12B	0.0507 (14)	0.1058 (19)	0.0449 (13)	−0.0032 (13)	0.0173 (11)	0.0041 (12)
C13B	0.0486 (13)	0.0741 (15)	0.0409 (12)	0.0076 (11)	0.0083 (10)	0.0022 (10)
C14B	0.0481 (13)	0.0926 (18)	0.0444 (12)	−0.0005 (12)	0.0137 (11)	−0.0012 (11)
C15B	0.0446 (12)	0.0875 (17)	0.0426 (12)	−0.0035 (11)	0.0069 (10)	0.0025 (11)
C16B	0.0604 (15)	0.0727 (16)	0.0593 (15)	0.0090 (12)	0.0022 (12)	0.0038 (12)
C17B	0.0601 (14)	0.0746 (16)	0.0561 (14)	−0.0014 (12)	0.0067 (12)	−0.0092 (12)
C18B	0.0507 (13)	0.0717 (15)	0.0484 (13)	−0.0107 (11)	0.0023 (11)	0.0071 (11)
C19B	0.0586 (14)	0.0637 (14)	0.0648 (16)	0.0007 (11)	0.0056 (12)	0.0063 (12)
C20B	0.0606 (15)	0.0724 (16)	0.0572 (15)	−0.0012 (12)	0.0097 (12)	−0.0074 (12)

### Geometric parameters (Å, º)

**Table:**

O1A—C13A	1.357 (2)	O1B—C13B	1.357 (3)
O1A—H1A	0.8200	O1B—H1B	0.8200
O2A—C14A	1.205 (2)	O2B—C14B	1.195 (3)
O3A—C14A	1.354 (2)	O3B—C14B	1.352 (3)
O3A—C15A	1.399 (2)	O3B—C15B	1.399 (3)
O4A—N6A	1.201 (3)	O4B—N6B	1.209 (3)
O5A—N6A	1.204 (3)	O5B—N6B	1.214 (3)
N6A—C18A	1.465 (3)	N6B—C18B	1.470 (3)
C7A—C8A	1.501 (3)	C7B—C8B	1.505 (3)
C7A—H7A1	0.9600	C7B—H7B1	0.9600
C7A—H7A2	0.9600	C7B—H7B2	0.9600
C7A—H7A3	0.9600	C7B—H7B3	0.9600
C8A—C9A	1.377 (3)	C8B—C9B	1.374 (3)
C8A—C13A	1.396 (3)	C8B—C13B	1.390 (3)
C9A—C10A	1.390 (3)	C9B—C10B	1.389 (3)
C9A—H9A	0.9300	C9B—H9B	0.9300
C10A—C11A	1.388 (3)	C10B—C11B	1.384 (3)
C10A—C14A	1.459 (3)	C10B—C14B	1.454 (3)
C11A—C12A	1.371 (3)	C11B—C12B	1.367 (3)
C11A—H11A	0.9300	C11B—H11B	0.9300
C12A—C13A	1.385 (3)	C12B—C13B	1.377 (3)
C12A—H12A	0.9300	C12B—H12B	0.9300
C15A—C16A	1.369 (3)	C15B—C20B	1.369 (3)
C15A—C20A	1.370 (3)	C15B—C16B	1.374 (3)
C16A—C17A	1.381 (3)	C16B—C17B	1.378 (3)
C16A—H16A	0.9300	C16B—H16B	0.9300
C17A—C18A	1.373 (3)	C17B—C18B	1.376 (3)
C17A—H17A	0.9300	C17B—H17B	0.9300
C18A—C19A	1.370 (3)	C18B—C19B	1.367 (3)
C19A—C20A	1.378 (3)	C19B—C20B	1.379 (3)
C19A—H19A	0.9300	C19B—H19B	0.9300
C20A—H20A	0.9300	C20B—H20B	0.9300
			
C13A—O1A—H1A	109.5	C13B—O1B—H1B	109.5
C14A—O3A—C15A	116.73 (15)	C14B—O3B—C15B	117.38 (17)
O4A—N6A—O5A	122.6 (2)	O4B—N6B—O5B	123.4 (2)
O4A—N6A—C18A	119.2 (2)	O4B—N6B—C18B	117.7 (3)
O5A—N6A—C18A	118.2 (2)	O5B—N6B—C18B	118.9 (2)
C8A—C7A—H7A1	109.5	C8B—C7B—H7B1	109.5
C8A—C7A—H7A2	109.5	C8B—C7B—H7B2	109.5
H7A1—C7A—H7A2	109.5	H7B1—C7B—H7B2	109.5
C8A—C7A—H7A3	109.5	C8B—C7B—H7B3	109.5
H7A1—C7A—H7A3	109.5	H7B1—C7B—H7B3	109.5
H7A2—C7A—H7A3	109.5	H7B2—C7B—H7B3	109.5
C9A—C8A—C13A	117.47 (17)	C9B—C8B—C13B	117.3 (2)
C9A—C8A—C7A	122.07 (17)	C9B—C8B—C7B	122.7 (2)
C13A—C8A—C7A	120.43 (17)	C13B—C8B—C7B	120.0 (2)
C8A—C9A—C10A	122.14 (17)	C8B—C9B—C10B	122.36 (19)
C8A—C9A—H9A	118.9	C8B—C9B—H9B	118.8
C10A—C9A—H9A	118.9	C10B—C9B—H9B	118.8
C11A—C10A—C9A	118.95 (17)	C11B—C10B—C9B	118.8 (2)
C11A—C10A—C14A	122.36 (17)	C11B—C10B—C14B	122.1 (2)
C9A—C10A—C14A	118.62 (16)	C9B—C10B—C14B	119.12 (19)
C12A—C11A—C10A	120.06 (18)	C12B—C11B—C10B	119.9 (2)
C12A—C11A—H11A	120.0	C12B—C11B—H11B	120.1
C10A—C11A—H11A	120.0	C10B—C11B—H11B	120.1
C11A—C12A—C13A	120.17 (17)	C11B—C12B—C13B	120.5 (2)
C11A—C12A—H12A	119.9	C11B—C12B—H12B	119.8
C13A—C12A—H12A	119.9	C13B—C12B—H12B	119.8
O1A—C13A—C12A	122.05 (17)	O1B—C13B—C12B	122.19 (19)
O1A—C13A—C8A	116.87 (17)	O1B—C13B—C8B	116.57 (19)
C12A—C13A—C8A	121.08 (17)	C12B—C13B—C8B	121.2 (2)
O2A—C14A—O3A	120.82 (17)	O2B—C14B—O3B	120.8 (2)
O2A—C14A—C10A	126.02 (18)	O2B—C14B—C10B	125.9 (2)
O3A—C14A—C10A	113.15 (16)	O3B—C14B—C10B	113.31 (18)
C16A—C15A—C20A	122.09 (19)	C20B—C15B—C16B	122.1 (2)
C16A—C15A—O3A	118.98 (18)	C20B—C15B—O3B	118.5 (2)
C20A—C15A—O3A	118.86 (19)	C16B—C15B—O3B	119.3 (2)
C15A—C16A—C17A	119.05 (19)	C15B—C16B—C17B	118.8 (2)
C15A—C16A—H16A	120.5	C15B—C16B—H16B	120.6
C17A—C16A—H16A	120.5	C17B—C16B—H16B	120.6
C18A—C17A—C16A	118.60 (19)	C18B—C17B—C16B	118.6 (2)
C18A—C17A—H17A	120.7	C18B—C17B—H17B	120.7
C16A—C17A—H17A	120.7	C16B—C17B—H17B	120.7
C19A—C18A—C17A	122.41 (19)	C19B—C18B—C17B	122.6 (2)
C19A—C18A—N6A	118.49 (18)	C19B—C18B—N6B	118.6 (2)
C17A—C18A—N6A	119.10 (19)	C17B—C18B—N6B	118.8 (2)
C18A—C19A—C20A	118.71 (19)	C18B—C19B—C20B	118.5 (2)
C18A—C19A—H19A	120.6	C18B—C19B—H19B	120.8
C20A—C19A—H19A	120.6	C20B—C19B—H19B	120.8
C15A—C20A—C19A	119.1 (2)	C15B—C20B—C19B	119.3 (2)
C15A—C20A—H20A	120.4	C15B—C20B—H20B	120.4
C19A—C20A—H20A	120.4	C19B—C20B—H20B	120.4

### Hydrogen-bond geometry (Å, º)

**Table:**

*D*—H···*A*	*D*—H	H···*A*	*D*···*A*	*D*—H···*A*
O1*A*—H1*A*···O2*A*^i^	0.82	1.94	2.753 (2)	172
O1*B*—H1*B*···O2*B*^ii^	0.82	1.94	2.727 (3)	160
C7*B*—H7*B*1···O4*A*^iii^	0.96	2.50	3.418 (3)	159
C12*A*—H12*A*···O2*A*^i^	0.93	2.54	3.245 (2)	132
C19*B*—H19*B*···O5*A*^iv^	0.93	2.58	3.476 (4)	163

Symmetry codes: (i) *x*, *y*−1, *z*; (ii) *x*, −*y*+1, *z*+1/2; (iii) −*x*+1/2, −*y*+3/2, −*z*+1; (iv) *x*, −*y*+2, *z*−1/2.
